# Transmission parameters estimated for *Salmonella* typhimurium in swine using susceptible-infectious-resistant models and a Bayesian approach

**DOI:** 10.1186/1746-6148-10-101

**Published:** 2014-04-28

**Authors:** Carla Correia-Gomes, Theodoros Economou, Trevor Bailey, Pavel Brazdil, Lis Alban, João Niza-Ribeiro

**Affiliations:** 1 ICBAS-UP - Department of Population Studies, Instituto de Ciências Biomédicas Abel Salazar – Universidade do Porto, Rua de Jorge Viterbo Ferreira, n°228, 4050-313 Porto, Portugal; 2 CEMPS, University of Exeter, Harrison Building, North Park Road, Exeter, EX4 4QF, UK; 3 LIAAD – INESC Porto L.A. Universidade do Porto, Rua de Ceuta, 118, 6, 4050-190 Porto, Portugal; 4 FEP-UP – Faculty of Economics – Universidade do Porto, Rua Dr. Roberto Frias, 4200-464 Porto, Portugal; 5 DMA - Danish Agriculture and Food Council, Axelborg, Axeltorv 3, DK-1609, Copenhagen, Denmark; 6 ISPUP – Institute of Public Health – Universidade do Porto, Rua das Taipas, 4099-003 Porto, Portugal; 7 ERU-SRUC – Epidemiology Research Unit, Future Farming Systems, SRUC, Drummondhill, Inverness, IV2 4JZ, UK

**Keywords:** *Salmonella* typhimurium, Transmission parameters, Bayesian approach

## Abstract

**Background:**

Transmission models can aid understanding of disease dynamics and are useful in testing the efficiency of control measures. The aim of this study was to formulate an appropriate stochastic Susceptible-Infectious-Resistant/Carrier (SIR) model for *Salmonella* Typhimurium in pigs and thus estimate the transmission parameters between states.

**Results:**

The transmission parameters were estimated using data from a longitudinal study of three Danish farrow-to-finish pig herds known to be infected. A Bayesian model framework was proposed, which comprised Binomial components for the transition from susceptible to infectious and from infectious to carrier; and a Poisson component for carrier to infectious. Cohort random effects were incorporated into these models to allow for unobserved cohort-specific variables as well as unobserved sources of transmission, thus enabling a more realistic estimation of the transmission parameters. In the case of the transition from susceptible to infectious, the cohort random effects were also time varying. The number of infectious pigs not detected by the parallel testing was treated as unknown, and the probability of non-detection was estimated using information about the sensitivity and specificity of the bacteriological and serological tests. The estimate of the transmission rate from susceptible to infectious was 0.33 [0.06, 1.52], from infectious to carrier was 0.18 [0.14, 0.23] and from carrier to infectious was 0.01 [0.0001, 0.04]. The estimate for the basic reproduction ration (*R*_*0*_) was 1.91 [0.78, 5.24]. The probability of non-detection was estimated to be 0.18 [0.12, 0.25].

**Conclusions:**

The proposed framework for stochastic SIR models was successfully implemented to estimate transmission rate parameters for *Salmonella* Typhimurium in swine field data. *R*_*0*_ was 1.91, implying that there was dissemination of the infection within pigs of the same cohort. There was significant temporal-cohort variability, especially at the susceptible to infectious stage. The model adequately fitted the data, allowing for both observed and unobserved sources of uncertainty (cohort effects, diagnostic test sensitivity), so leading to more reliable estimates of transmission parameters.

## Background

*Salmonella* Typhimurium is one of the major food-borne pathogens currently causing disease in humans [1] and it is often related to consumption of pork products. Given its relevance to consumer food safety, *Salmonella spp*. control was considered necessary by the European food safety policy makers under the EC Regulation 2160/2003. In the near future, it is possible that a mandatory target reduction will be put in place in the European Union, regarding the *Salmonella* prevalence for pigs.

In practice, however, the control of this agent has proved to be difficult and expensive at the farm level [2]. Consequently, evaluating the efficiency of control strategies relating to this agent has become an important issue, as stated in recent reports [3]. Modelling the dynamics of *Salmonella* Typhimurium in pigs is important in evaluating alternative control strategies. The basic reproduction ratio (R_0_) expresses the number of secondary cases to which a primary case gives rise during the infectious period and provides valuable information for simulation models. If R_0_ is less than unity then the disease is receding, but when it is higher than unity the disease is spreading.

Susceptible – Infectious – Resistant (SIR) models are attractive tools that aid understanding of disease dynamics. The SIR model formulates the changes of individuals in the population between different disease states in terms of a system of ordinary differential equations (ODE), known as the Kermack-McKendrick ODE model [4]. The variables in the system are given by the three components groups: susceptible (S), infectious (I) and carriers (R). SIR models include a mathematical specification of the movement into and out of the three components. The key parameter in each of these mathematical specifications is the transition rate: from S to I (β), from I to R (α) and from R to I (ν). If such modelling is to be helpful in infectious disease control, it is critical to have the best possible estimates of these rates (β, α and ν), as all three are important for modelling the spread of infection. Transmission data, generated under controlled conditions (i.e. experimental studies or controlled field studies) are necessary to estimate the transition rates as accurately as possible.

In most cases *Salmonella* Typhimurium causes subclinical infection in swine with no apparent symptoms of disease. This makes it difficult to assess the infection status of individual pigs in an infected population without testing each animal several times. One of the difficulties in obtaining accurate estimates for β in *Salmonella* Typhimurium studies stems from the fact that the currently available bacteriological and serological tests used to assign the infection status are imperfect, introducing uncertainty when trying to classify each animal. Yet another source of uncertainty comes from the fact that pigs, once infected, shed the agent intermittently.

In the literature, it is conventional to use generalised linear models (GLMs) to describe the counts of animals moving between states (e.g. from S to I) using either Poisson [5-8] or Binomial distributions [4,9]. GLMs can be used to estimate all three transmission parameters although they lack flexibility, for example in capturing the effect of sensitivity and specificity of the diagnostic test used. GLMs also lack the flexibility to allow for unobserved effects of variables not recorded in the data, but which influence the outcomes. The Bayesian framework proposed in this paper is flexible enough to incorporate such effects and also quantify the uncertainty due to imperfect diagnostic tests.

Following cohorts of animals in order to determine the dynamics of *S*. Typhimurium in susceptible populations is a very expensive procedure and only a few such studies exist. In this paper, we use data from a previous observational study designed and performed by Kranker *et al.*[10].

A Bayesian modelling framework was proposed and used to estimate transmission parameters (transition rate from S to I, transition rate from I to R and transition rate from R to I) for *Salmonella* Typhimurium in pig herds, using the longitudinal data from Kranker *et al.*[10]. The sensitivity and specificity of the tests used to classify the animals in the Kranker study were allowed for in the statistical model, which also incorporated random effects to allow for cohort heterogeneity.

## Methods

### Study herds, sampling, bacteriology and ELISA test

The data used have been previously described by Kranker *et al.*[10] and originate from three Danish pig herds known to be infected with *Salmonella* Typhimurium. The herds had moderate to high levels of *Salmonella* Typhimurium and therefore the within-herd prevalence was 40% or higher. These measures of prevalence were based on meat-juice samples collected over three months, evaluated by use of an optical density (OD) cut-off of 20%. Two of the farms, with 650 and 440 sows, respectively, were two-site operations; the remaining farm was a three-site operation with 300 sows. The three herds were self-supplying. In each herd, 10 litters were randomly selected, and in each litter, the ears of six randomly selected piglets were tagged. To account for variations in *Salmonella* shedding over time, litters from each herd were divided into two groups of five litters that were raised at approximately one-month intervals. Thus, on each farm there were two cohorts consisting of 30 pigs each, yielding a total of 180 piglets at the start of the study. All ear-tagged pigs from a given cohort were raised together for the entire observation period. The animals were followed longitudinally [10] and were first tested at the age of four weeks and thereafter at two to five week intervals until the age of slaughter. Slaughter age varied between cohorts but was on average around 25 weeks. Cohorts were tested either six to seven times in total. On each testing occasion, sera and faeces from the animals were collected and tested for the presence of *Salmonella spp*.. At the age of four weeks only faeces were collected, because maternal antibodies still present could give a false positive result. An animal was considered serologically positive, wherever the serological test revealed a result of OD% >20, and bacteriologically positive if *Salmonella* was isolated from the faeces. The serological test used at this cut-off value is considered to have a sensitivity of 68% and to be 100% specific [11]. The bacteriological test is considered to be 100% specific and the sensitivity is around 30 to 55% [12]. These test characteristics were incorporated in the statistical model.

### Infection status of the pigs

The testing time interval was different in each cohort, varying from two to five weeks. A homogenous dataset was derived by inferring the infection status of each pig, every two weeks. The time step of two weeks was chosen because on average it takes two weeks for an animal to test positive to serology after being infected. It was therefore assumed that an animal was infectious in the two weeks before being seropositive. The most likely infection status of each pig was determined for each two-week time step based on both faecal shedding and serology results from the sampling period closest to each time step. Each animal was categorized as susceptible (S), infectious (I) or carrier (R). An animal was considered susceptible if the agent was not present and the animal was at the risk of infection. An animal was considered infectious if it was shedding the agent and a potential source of infection to other animals. An animal that was infected with the agent but not shedding and therefore not able to infect other animals was considered a carrier. In the absence of reasonable sensitivity of the bacteriological culture method, serology offered an alternative and complementary way to assign the infection status of a pig and both methods were used in parallel to categorise the pigs’ status.

Pigs were attributed status S when there was no presence of bacteria in the faecal samples and the OD% was below 20. Status I was assigned from the date when a pig was found to be bacteriologically positive until it was no longer positive by this testing method. Additionally, pigs were assigned to status I based on seroconversion. The beginning of the infectious period was set to two weeks prior to the recorded date of seroconversion [13,14] and the duration was set to four weeks, assuming that a pig would shed *Salmonella spp*. within an average of four weeks. This average period was based on experimental data regarding duration of the shedding period [13,15]. Thus, information was used from both tests in parallel for pig classification. Finally, status I was followed by status R and the pigs could return to status I if they were found to be culture positive later on during the study period. It was assumed that no pig would return to the susceptible status after being infected, because of the relative short life span of finisher pigs (after infection it takes around 112 days to clear the agent from the organs [14], and post-weaned pigs are generally slaughtered before this time). The following describes a particular example of how the classification was performed: if a pig was shedding at a specific testing time, it was considered infectious in the nearest bi-weekly time step, until it became bacteriologically negative, after which it was considered a carrier (in the nearest bi-weekly time step). If a pig was serologically positive, in the presence of a negative culture, it was considered infected and therefore classified as infectious for at least four weeks, from the nearest bi-weekly time step prior the testing time. If an animal was bacteriologically and serologically negative, it was considered susceptible. Given that testing of piglets was restricted to bacteriology (which has low sensitivity) at the beginning of the follow-up period, some piglets infected by the sow could have been erroneously classified as susceptible. For this reason, the analysis in each cohort started at the time infected animals were first detected (by either serology or bacteriology).

### Estimation of the transmission parameters

Conventionally, transmission parameters of infectious disease, including *Salmonella spp*., in swine herds [14,16-21] are estimated using regression models. These are often based on data describing the prevalence of that disease in the country or region to which the particular study refers. As suggested in some studies [5,22,23], we first applied a stochastic SIR models in the form of Generalised Linear Models (GLMs) in order to estimate the three transmission parameters. However, preliminary results (not reported here) suggested the presence of overdispersion in the GLMs, hinting towards unobserved sources of variation in the data such as cohort heterogeneity. Instead, we here report a framework for stochastic SIR models which i) extends the current GLM framework by including random effects, ii) is implemented using a Bayesian approach thus allowing incorporation of prior information (such as the sensitivity of *Salmonella* tests), iii) explicitly estimates the probability of not detecting infectious animals due to test sensitivity and iv) incorporates all sources of uncertainty/variation thus obtaining more realistic estimates of transmission parameters. As suggested by some authors [5], the inclusion of random effects automatically accounts for overdispersion by inflating the variance of the response variable while at the same time allowing for cohort heterogeneity.

Stochastic SIR models (and other variants such as SI or SIS models) are well-established in animal disease literature [4,19-21]. The benefit of using a stochastic SIR model is that transmission parameters can be estimated using statistical modelling; here the conventional stochastic SIR model was extended by explicitly allowing for cohort variation and unobserved temporal effects. The three components of the stochastic SIR model are described in detail below.

### Transition from susceptible to infectious

It was assumed that pigs become infected by “infectious contacts” defined as: either contact with other infected animals, or contact with their environment (rodents, contaminated muck or feed). The rate at which a given animal has infectious contacts was assumed i) to be constant in time and ii) proportional to the density of infectious animals [19], with a constant of proportionality β, i.e. the transmission rate parameter. In other words, the infectious contacts per animal happen randomly in time so that their occurrence can be described by a Poisson process. More precisely, the number of infectious contacts per animal in a period Δt is Poisson distributed with mean λ = β (I/N) Δt, where I is the number of infectious animals and N is the total number of animals, at the beginning of Δt. As such, the probability of no infectious contacts per animal in Δt is exp (-β (I/N) Δt), implying that the probability of infection in Δt is p = 1- exp (-β (I/N) Δt). This means that the number of new cases C at the end of Δt is Binomial with parameters S and p so that the mean of C is S*p.

Here, the current established methodology was extended to allow for the fact that i) λ may vary in time due to exogenous factors and ii) λ may vary across cohorts due to unobserved cohort effects. A random (scaling) effect exp (r_jt_) was included, for the j^th^ cohort at time t, to get λ_jt_ = β (I/N) exp (r_jt_) Δt as the mean number of infectious contacts of a random animal, in herd j at time t. Note that Δt denotes the length of a time interval whereas t refers to actual time. On average, exp (r_jt_) was assumed to be equal to one, so that across all cohorts and time, the average transmission rate parameter is still β. By doing this, variation due to cohort or unknown temporal effects was explicitly modelled, which would otherwise contribute to the uncertainty in estimating β.

All time intervals in the data are equal to two weeks so for clarity, Δt = 1 was set so that one time step Δt corresponds to two weeks. This does not qualitatively affect the estimation of the transmission parameters. Because of the nature of the data, time t is now defined in discrete consecutive (bi-weekly) time steps.

The model may be formulated as follows:

(1) $$\begin{array}{l} C_{\mathrm{jt}}\sim \mathrm{Binomial}\left(S_{\mathrm{jt}},p_{\mathrm{jt}}\right)\\ p_{\mathrm{jt}}=1–\exp\left\{-\beta \left(I_{\mathrm{jt}-1}/N_{\mathrm{jt}-1}\right)\exp\left(r_{1\mathrm{jt}}\right)\right\}\\ \mathrm{cloglog}\left(p_{\mathrm{jt}}\right)=\log\left(\beta \right)+\log\left(I_{\mathrm{jt}-1}\right)-\log\left(N_{\mathrm{jt}-1}\right)+r_{1\mathrm{jt}} \end{array}$$

where:

– C_*jt*_ denotes the number of new infectious animals in cohort (j) at the end of the time step (t),.

– S_*jt*-*1*_ is the number of susceptible animals in cohort (j) at the end of the time step (t-1),

– p_jt_ is the probability of a susceptible animal in cohort (j) at the end of time step (t-1) becoming infectious by the end of time step (t),

– cloglog is the complementary log-log transformation,

– β is the transmission rate parameter for the transition from susceptible to infectious,

– I_*jt*-*1*_ is the number of infectious animals in cohort (j) at the end of the time step (t-1),

– N_*jt*-*1*_ is the total number of animals in cohort (j) at the end of the time step (t-1), and.

– r_1*jt*_ is a cohort time-dependent random effect (which is zero on average).

Note that, at the beginning of the study, pigs were considered to be either in the S or I status depending on the test results. When there was no infectious pig present at the end of the previous time step, i.e. I_jt-1_ = 0, the probability of becoming infectious was modelled as:

Cloglog (p_jt_) = log (β) + r_1jt_. This is because even if there are no infectious pigs present, animals can still be infected (e.g., contaminated environment, feed, water, etc.). In this formulation, β is seen as the underlying rate of transition for a random pig in an average cohort with no infectious animals, while r_1jt_ allows for unobserved cohort-time effects in the data e.g., anthropogenic influence, rodents etc. Note that homogeneous mixing of the pigs in each cohort (i.e. all pigs could come into contact with each other) was assumed, due to the small size of the cohorts.

In using the number of infectious pigs I_jt_, in each cohort at the end of time step t, it was necessary to account for the sensitivity of both the serological and bacteriological test. Since the specificity in both tests is considered to be 100%, the parallel specificity is 1. This implies that I_jt_ = Iobs_jt_ + Inob_jt_, where Iobs_jt_ is the observed value and Inob_jt_ is the number of infectious animals not detected (false negative pigs). In other words, Iobs_jt_ is a lower bound on the actual I_jt_. The unobserved variable Inob_jt_ may be incorporated (and thus estimated) in the stochastic model and here it was assumed that it has a Binomial distribution with parameters N_jt_ and pND where pND is the probability of not detecting infectious animals. This probability, pND, is of course dependent on the sensitivity probabilities of each test, which were assumed to be independent. Inob_jt_ was modelled as follows:

(2) $$\begin{array}{l} I_{\mathrm{jt}}={\mathrm{Iobs}}_{\mathrm{jt}}+{\mathrm{Inob}}_{\mathrm{jt}}\\ {\mathrm{Inob}}_{\mathrm{jt}}\sim \mathrm{Binomial}\left(N_{\mathrm{jt}},\mathrm{pND}\right)\\ \mathrm{pND}=\left(1-\mathrm{SenC}\right)*\left(1-\mathrm{SenE}\right) \end{array}$$

where:

– *SenC* is the sensitivity probability of microbiological culture, and.

– *SenE* is the sensitivity probability of the ELISA test.

Treating Inob_jt_ as an unobserved random variable allows formal quantification of the uncertainty in the data due to test sensitivity and constitutes one of the novelties of the proposed model. The Bayesian framework (see section 5 later on) used to estimate the stochastic SIR model can easily incorporate the estimation of Inob_jt_ given prior information on SenC and SenE.

### Transition from infectious (I) to resistant (R)

The rate α at which a random infectious animal in a given cohort becomes a carrier was assumed to be constant in time. As such, the length of time τ until an infectious animal becomes carrier can be modelled by an exponential distribution with rate parameter α. So, given that the animal is infectious at the start of time interval Δt, the probability pR of becoming carrier is pR = Pr (τ ≤ Δt) = 1-exp (-αΔt) since τ is exponentially distributed (recall that Δt = 1 was set for conciseness). As before, a random cohort effect r_2j_ was added to allow for cohort heterogeneity in the data, to obtain pR_j_ = 1-exp (-αexp (r2_j_)). The number of new carrier animals Rnew_jt_ at the end of time step t, is thus Binomial with parameters I_jt_ and pR_j_. Note that a single parameter α was utilised, describing the rate at which a random infectious animal in an average cohort, becomes carrier, however, cohort variability (not all cohorts are average) was allowed for through r_2j_, which in turn reduces uncertainty in estimating α. The I to R transition was modelled as follows:

(3) $$\begin{array}{l} {\mathrm{Rnew}}_{\mathrm{jt}}\sim \mathrm{Binomial}\left(I_{\mathrm{jt}},{\mathrm{pR}}_{j}\right)\\ \mathrm{cloglog}\left({\mathrm{pR}}_{j}\right)=\log\left(\alpha \right)+r_{2j} \end{array}$$

### Transition from resistant to infectious

For this compartment of the model, the rate of infectious contacts ν in a random carrier animal was assumed to be constant in time, where ν is the transmission rate parameter for the transition from carrier to infectious. With similar arguments as in the S to I compartment, the number of infectious contacts per animal in time period Δt is Poisson distributed with mean νΔt. Since this transition was actually a rare event (only happening three times in the entire study), the Poisson distribution can be used, as it approximates the Binomial when its probability parameter is close to zero. So if in cohort j, there are R_jt-1_ carrier animals at the end of the previous time step, the number of transitions from R to I in time step t may be modelled as a Poisson variable with mean μ_jt_ = νR_jt-1_exp (r_3j_). More explicitly:

(4) $$\begin{array}{l} {\mathrm{Inew}}_{\mathrm{jt}}\sim \mathrm{Poisson}\left(\mu_{\mathrm{jt}}\right)\\ \log\left(\mu_{\mathrm{jt}}\right)=\log\left(\nu \right)+\log\left(R_{\mathrm{jt}-1}\right)+r_{3j} \end{array}$$

where:

– Inew_jt_ denotes the number of new infectious animals (that result from this transition) in cohort (j) at the end of the time step (t),

– μ_jt_ is the mean number of carrier animals that become infectious in the cohort (j) during time step (t),

– *ν* is the transmission rate parameter for the transition from carrier to infectious state,

– R_jt-1_ is the number of carrier animals at the end of the time step (t-1) in cohort (j), and.

– r_3*j*_ is a cohort random effect that allows for cohort heterogeneity.

Note that R_jt-1_ = 0 is possible, in which case log (R_jt-1_) = 0 was set. The argument for doing this is that the transmission rate parameter ν may be defined as the limit of μ_jt_/R_jt-1_as R_jt-1_ goes to zero. As such, ignoring the random effect for a moment, μ_jt_/R_jt-1_ should tend to a constant (i.e. ν) as R_jt-1_ goes to zero rather than infinity. Note that in our data, R_jt-1_ = 0 happened on 20% of occasions. In the hypothetical case that R_jt-1_ = 0 for the majority of time steps and cohorts, then this component of the model (i.e. the transition R to I) becomes redundant as there will ultimately be almost no information with which to estimate the transition parameter.

### Cohort random effects

As indicated above, random cohort effects were incorporated into each transition step to allow for i) cohort heterogeneity/variability in the data, ii) unobserved cohort-specific factors, iii) unobserved temporal effects in the S to I compartment. These effects were different for each transition under the assumption that any unobserved cohort factors affect each transition in a different way. For the transitions S to I and R to I, these random effects also allow for factors which affect disease spread but which are not dependent on the animals themselves (such as contaminated environment, feed, water, etc.).

For the transition S to I, the cohort random effects were assumed to be time-varying and auto-correlated, and were modelled as:

(5) $$\begin{array}{l} r_{1j,t=1}\sim \mathrm{Normal}\left(0,\sigma_{1}^{2}\right)\\ r_{1j,t}\sim \mathrm{Normal}\left(r_{1j,t-1},\sigma_{1}^{2}\right) \end{array}$$

where the cohort random effect (r_1*jt*_) for time step t depends on the previous cohort random effect at time (t-1). With this cohort time-dependent random effect any unobserved dynamic behaviour in the spreading of infection within cohorts was captured, such as that due to infected mice.

For the transition I to R and R to I, the random effects were modelled as:

(6) $$r_{\mathrm{kj}}\sim \mathrm{Normal}\left(0,\sigma_{k}^{2}\right),k=2,3$$

where:

– subscript *j* denotes cohorts and.

– $\sigma_{k}^{2}$ is the variance of the unobserved cohorts effects.

In a preliminary model building stage, a cohort time-dependent random effect, r_2jt,_ was considered for the transition I to R; however the results showed no improvement to the model fit. Note that cohort time-dependent random effects were not considered for the Poisson model of the transition R to I. The transition only occurred three times in the entire study and it would be unreasonable to try to estimate unobserved temporal effects from this.

### Model implementation

The overall SIR model described above was implemented in a Bayesian framework and fitted using Markov chain Monte Carlo (MCMC). In this framework, parameters are treated as random variables whose “prior” distribution expresses our uncertainty about their value before any data is observed. Prior distributions (priors) are combined with the observed data through Bayes theorem to produce the posterior distributions for each parameter (posteriors). The posteriors express the uncertainty about model parameters after data is observed and all statistical inference is based solely on the posteriors. MCMC is a numerical technique which produces samples of values that eventually converge (after a certain “burn-in” number) to samples of values from the posterior (distribution) of each parameter.

There was no historical information with which to inform the prior distributions of log (β), log (α) and log (ν), so Normal distributions with zero mean and a variance of 100 were used, reflecting prior ignorance while avoiding the use of improper prior distributions [24]. For the sensitivity probabilities of both serological and bacteriological tests, a Beta distribution was used as a prior. Previous information about the sensitivity of both tests [11,12] was used to inform those Beta distributions: a mean of 0.49 for faecal culture and a mean of 0.68 for Danish mix ELISA were assumed, so SenC ~ Beta (48.5, 50.5) and SenE ~ Beta (58.5, 27.5) were specified. These priors have means 0.49 and 0.68 respectively, and variances that match the range of possible values dictated by the findings of [11,12]. Specificity was assumed to be 100% in both tests. The precision (i.e. the inverse of the variance) of the Normal distribution for each random effect was given a Gamma (0.5, 0.005) prior distribution (large mean and very large variance to indicate prior ignorance).

The complete SIR model was implemented in the open-source statistical software WinBUGS [25]. Exactly 100,000 posterior samples were collected after a 5,000 sample burn-in to ensure convergence to the posterior distribution [26]. Two MCMC runs were performed, with different initial values, to ensure convergence and mixing. The samples were thinned by only collecting one in 10 consecutive samples to eliminate autocorrelation in posterior samples (the R package “coda” [27] was used), so that in total we ended up with 20,000 samples. Convergence was assessed by inspection of trace-plots but also more formally using the Raftery and Lewis diagnostic, and the Gelman-Rubin R-hat diagnostic which should be sufficiently close to one if convergence has been achieved [28,29]. Mixing in the chains was assessed by comparing the Markov Chain (MC) error with the standard deviation, for each parameter. Ideally the MC error for each parameter should be less than 5% of the standard deviation [30] for good mixing.

Posterior predictive simulation was used for model checking as described by Gilks *et al*. [24]. This technique is effectively testing whether the observed data are extreme in relation to the posterior predictive distribution of the observations (i.e., the fitted model). The deviance was the measure adopted for comparison. The technique involves the calculation of a “p-value” which should not be extreme (close to 0 or 1) for good model fit.

### Calculations of the basic reproduction ratio (R_0_)

Samples from the posterior distribution of *R*_*0*_ were calculated from those of β and α using the following formula [5]:

(7) R0=βα

where β is the transition rate from S to I, and α is the transition rate from I to R.

## Results

### Transmission parameters

Results, in terms of summary statistics from the posterior samples, are shown in Table 1. Note that the posterior samples are MCMC samples from the posterior distribution of each model parameter and inference is based on those samples. A point estimate, the standard error and the 95% credible interval for a parameter are, for example, calculated as the sample mean, the sample standard deviation and the sample 2.5% and 97.5% quantiles of the posterior samples for that parameter.

**Table 1:** **Summary measures of the transmission parameters and random effects variances from the *Salmonella *** **transmission in pigs SIR model**

**Parameters**	**Mean**	**Standard deviation**	**Quartiles**					**Rhat**
			**2,5%**	**25%**	**50%**	**75%**	**97,5%**	
**β**	0.44	0.49	0.06	0.20	0.33	0.52	1.52	1.0021
**α**	0.18	0.02	0.14	0.17	0.18	0.20	0.23	1.0009
**ν**	0.02	0.03	0.0001	0.006	0.01	0.02	0.04	1.0009
$\sigma_{1}^{2}$	3.00	1.80	0.80	1.76	2.60	3.77	7.59	1.0011
$\sigma_{2}^{2}$	0.02	0.04	0.002	0.005	0.01	0.02	0.096	1.0009
$\sigma_{3}^{2}$	6.64	38.82	0.003	0.06	0.08	3.85	44.44	1.0010
**pND**	0.18	0.03	0.12	0.16	0.18	0.20	0.25	
**R**_**0**_	2.20	1.25	0.78	1.46	1.91	2.56	5.24	

Legend: β – transition rate from susceptible to infectious, α – transition rate from infectious to carrier, ν – transition rate from carrier to infectious, $\sigma_{1}^{2}$ – variance of the random effects for the transition from susceptible to infectious, $\sigma_{2}^{2}$ - variance of the random effects for the transition from infectious to carrier, $\sigma_{3}^{2}$ - variance of the random effects for the transition from carrier to infectious, *pND* – probability of non-detection of infectious animals, R_0_ – basic reproduction ratio.

The MCMC convergence was considered acceptable since the R-hat for all parameters (including random effects) was never above 1.01. The results of the model did not significantly differ when the parameters of the priors for the sensitivity tests were varied (increasing and decreasing them by 10%).

**Figure 1:**
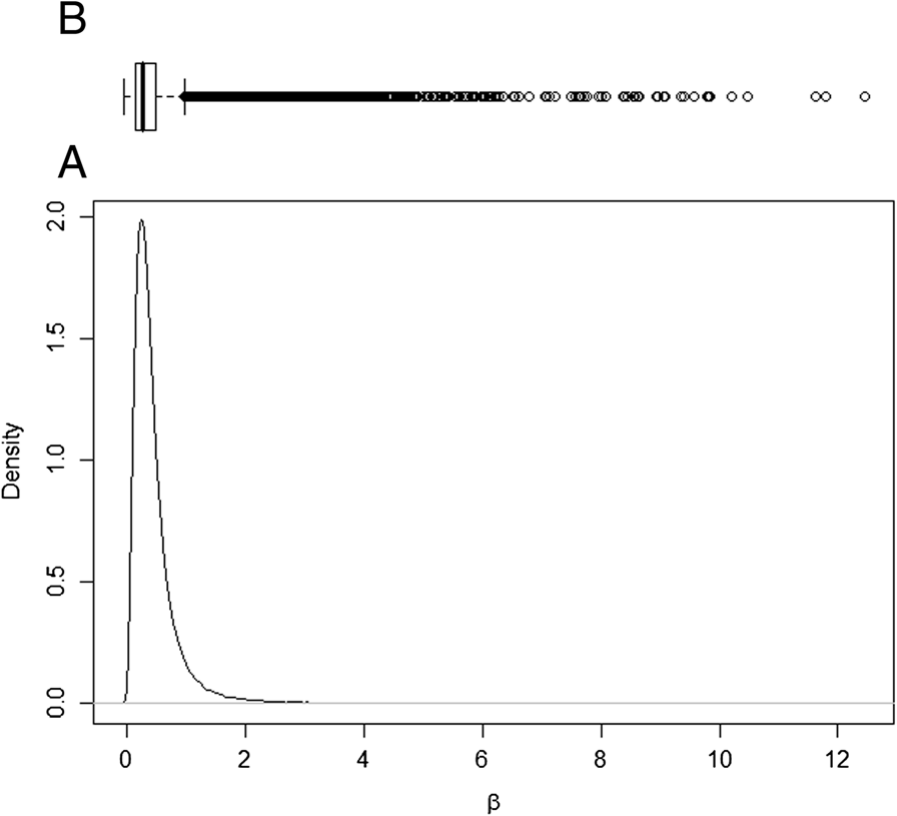
**Posterior distribution of β.** Legend: **A)** Plot of the posterior distribution for transmission parameter β, which describes the rate of spread of *Salmonella* Typhimurium from susceptible to infectious animals; **B)** Boxplot of the posterior samples used to produce the plot where the thick line in the box reflects the median.

**Figure 2:**
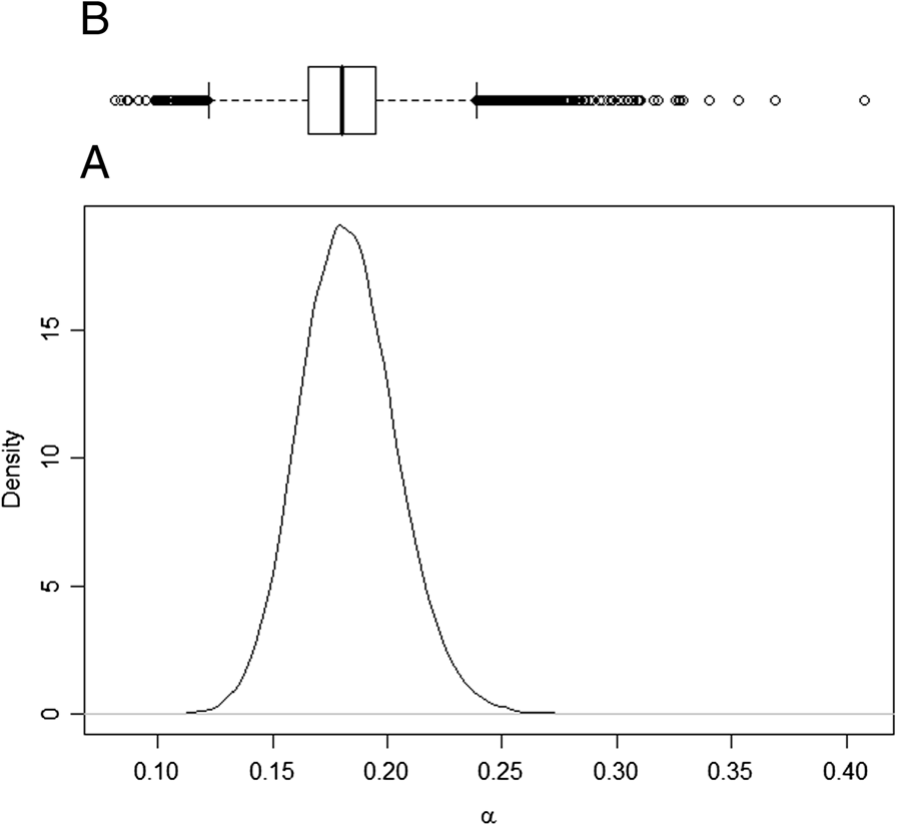
**Posterior distribution of α.** Legend: **A)** Plot of the posterior distribution for transmission parameter α, which describes the rate of spread of *Salmonella* Typhimurium from infectious to resistant animals; **B)** Boxplot of the posterior samples used to produce the plot where the thick line in the box reflects the median.

**Figure 3:**
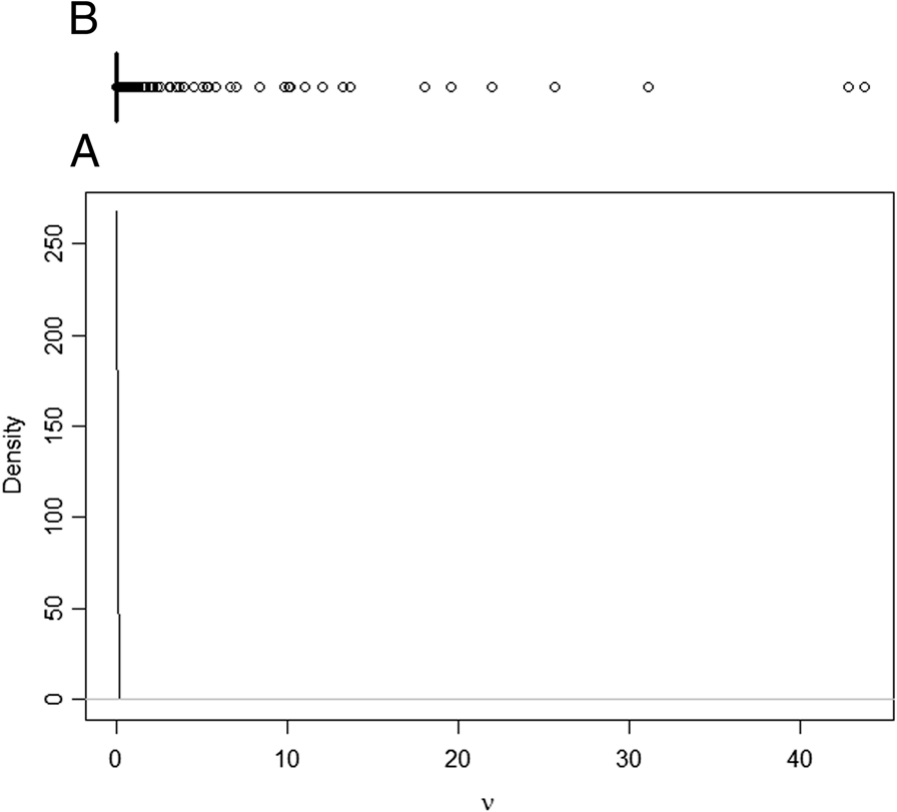
**Posterior distribution of ν.** Legend: **A)** Plot of the posterior distribution for transmission parameter ν, which describes the rate of spread of *Salmonella* Typhimurium from resistant to infectious animals; **B)** Boxplot of the posterior samples used to produce the plot where the thick line in the box reflects the median.

**Figure 4:**
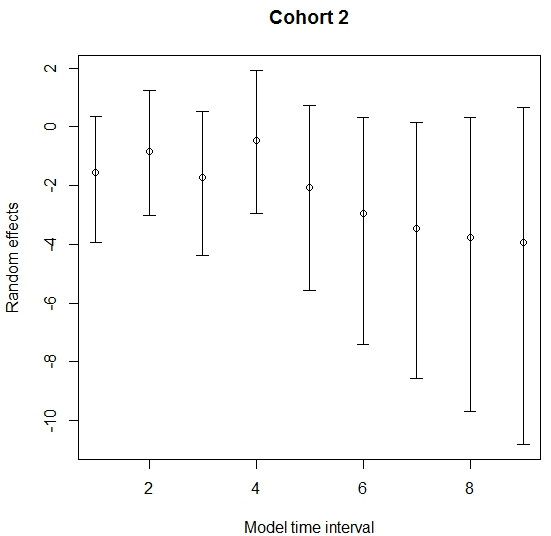
**Posterior distribution of the β random effects for cohort two over time.** Legend: Plot of the posterior distribution of the β random effects (time and cohort) for one cohort over time with the mean and 95% credible intervals.

The posterior distribution for transition rate α (I to R) was symmetric, but for the transition rate β (S to I) and ν (R to I), the posterior distributions were asymmetric (Figures 1, 2 and 3). As such, the posterior median was chosen to best summarise the value of these parameters. The median for the transition rate β was 0.33, for α it was 0.18 while for ν it was 0.01 (Table 1). The median of the variance of cohort random effects for the transitions I to R and R to I was close to zero, which implies that there was little significant variation between cohorts for these two transitions of the model. The median of the variance of the cohort-time dependent random effect for the transition S to I was 2.6 (95% credible interval [0.80; 7.59]), meaning that the cohort random effect is significant for this transition (Figure 4). The overall model fit was satisfactory with a “p-value” of 0.24 implying no significant difference between posterior predictive simulations (predictions from the model) and observed data.

### Basic reproductive ratio (R_0_)

**Figure 5:**
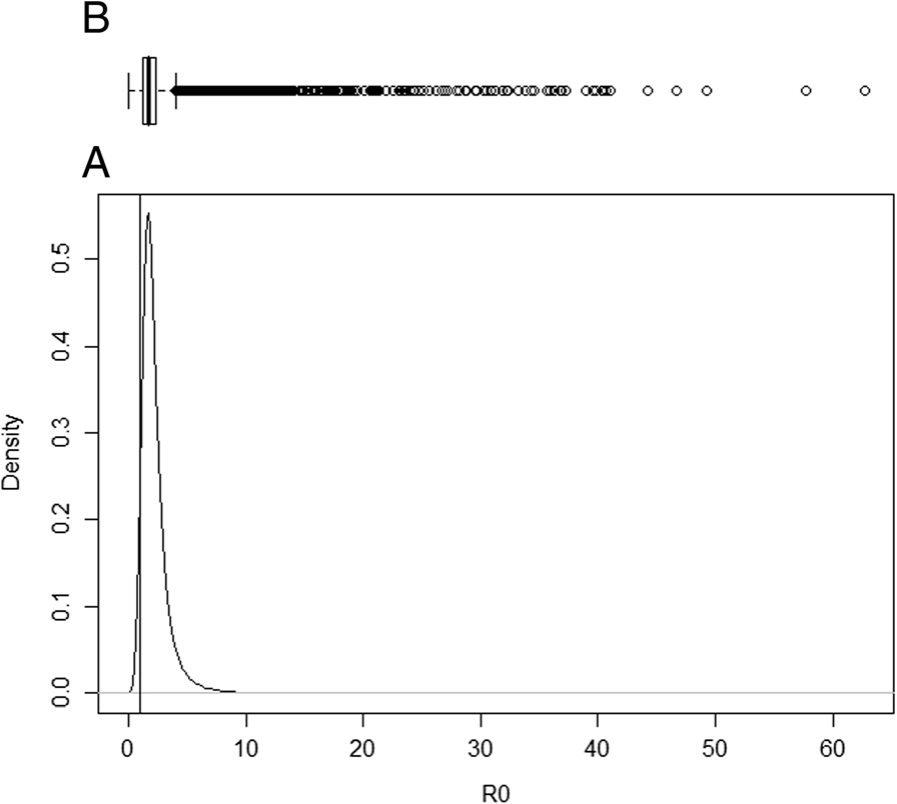
**Posterior distribution of the basic reproduction ratio (R0).** Legend: **A)** Plot of the posterior distribution for the basic reproduction ratio (R0). The vertical line shows the threshold value R0 = 1 where dissemination of the infection occurs; **B)** Boxplot of the posterior samples used to produce the plot where the thick line in the box reflects the median.

Summary statistics of the posterior distribution of the *R*_*0*_ parameter are shown in Table 1. The posterior median of *R*_*0*_ was 1.91, with a 95% credible interval of 0.78 to 5.24. A density estimate of the posterior samples of *R*_*0*_, which effectively describes the spread of *Salmonella spp*. in these three Danish pig herds known to be infected with *Salmonella*, is shown in Figure 5. For moderate to high within herd *Salmonella* prevalence, this *R*_*0*_ distribution suggests that *Salmonella* Typhimurim can range from fading out scenarios to epidemic ones but most of the time the infection spread assumes an endemic form.

### Diagnostic test sensitivity (pND)

**Figure 6:**
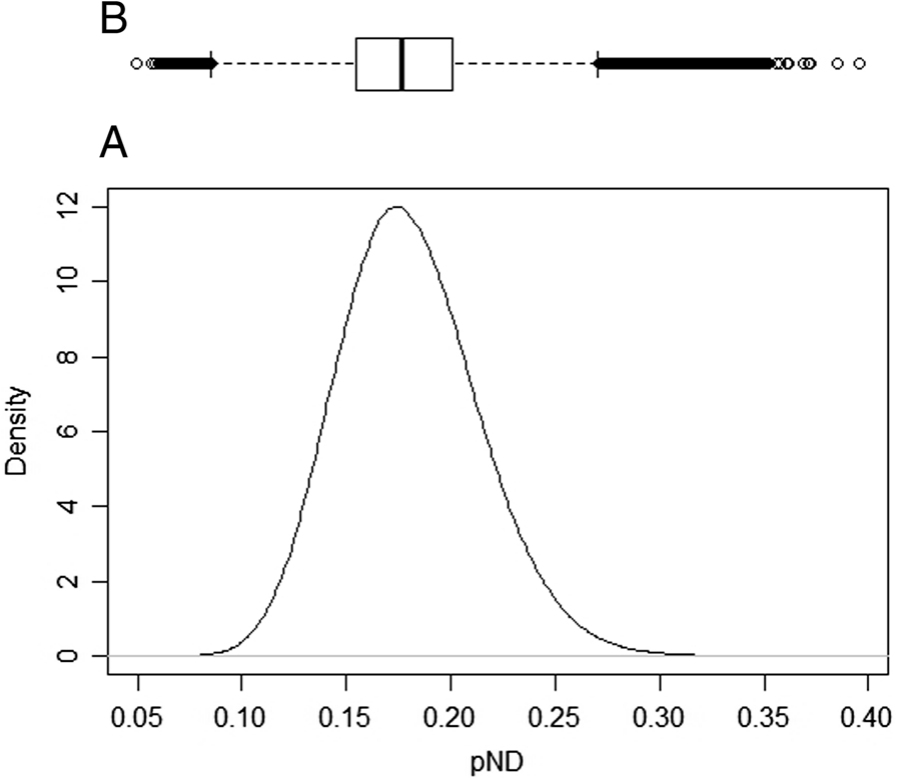
**Posterior distribution of the probability of non-detection of infected animals (*pND*).** Legend: **A)** Plot of the probability of non-detection of infected animals (*pND*) due to the test characteristics; **B)** Boxplot of the posterior samples used to produce the plot where the thick line in the box reflects the median.

Recall that this modelling framework includes the estimation of the probability of failing to detect infectious animals, pND, using both the data but also prior information about the tests sensitivity [11,12]. Figure 6 shows a density estimate plot of the posterior distribution of pND.

## Discussion

In this paper field data were used from a study conducted in three Danish pig herds which were known to be infected with *Salmonella* Typhimurium [10]. The data was considered sufficiently reliable to be used in estimating the transmission parameters of an SIR model describing the evolution of the disease and from that to infer *R*_*0*_ for *Salmonella* Typhimurium in finisher pigs.

In comparing the parameters to those found in other studies (whether simulation- or observation-based studies) it is important to take into consideration that the time interval used in this study was two weeks whereas in past simulation studies it varies from one day [14,17] to one week [18]. As the transmission parameters are rates, however, they can be easily transformed to relate to different time steps. Although the herds used in the Kranker study [10] had moderate to high levels of *Salmonella* Typhimurium prevalence, the median estimates of the transmission parameters from this study are lower than those found in previous simulation studies [16,18]. The transition rate β from S to I is slightly higher when compared to the Lurette *et al*. study [18] although the other rates (α, ν) are lower than the equivalent parameters in that same study. So the application of our proposed modelling framework to these Danish herds resulted in estimates comparable to similar studies (note that this approach could easily be used with data from other countries). Moreover, the prevalence of *Salmonella* in finishing pigs in Denmark is known to be close to the average prevalence in the EU [31].

To the best of our knowledge this is the first study of transmission rate parameters for *Salmonella* Typhimurium in swine that estimates the parameters using field data and a Bayesian probabilistic approach incorporating random effects.

### Bias of the study

Correct classification of the infectious status of the individual pig is difficult for *Salmonella* Typhimurium infection, because the diagnostic tests currently used are imperfect [32-37]. Bacteriology lacks sensitivity given intermittent shedding of *Salmonella* by infected pigs, whereas using serology in individuals can be associated more with a past exposure to the agent than a current exposure; therefore it can lack specificity for detecting shedding animals. Positive serology also shows a delay between infection and expression, leading to some lack of sensitivity. When analysing the data, the lack of sensitivity was accounted for by: i) starting the analysis when at least one infected pig per cohort was observed and ii) using the probabilistic framework to predict the infectious animals that were not detected with these tests, from appropriately informed distributions based on the sensitivity of each test.

For optimal estimation of transmission parameters, the time step between each sampling should preferably be as short as the average generation interval, spanning the time when one animal becomes infectious to the time when a second animal becomes infectious because of the first animal. The time steps in this data (two weeks) are not ideal – preferably a few days or perhaps one week would be better [13]. However as previously discussed, the available data did not allow for such an option and it would be very costly to obtain new data. As data from a published study [10] were used, the time step was set to be an approximation of the different testing intervals within and between cohorts, given the limitations offered by the original set of data, and an approximation to the time of seroconversion [13,38]. This approximation could have affected the estimation of parameters due to the extended time interval between testing occasions. Nevertheless, comparison with the results published in other studies does not seem to support this hypothesis. Concerning the cohorts, it is clear from the Kranker study [10] that particular attention was paid to the selection of the herds, which were taken from a large population of Danish finishing herds with a well-known status for *Salmonella*. This gave us confidence regarding the generalization of our results, at least for herd infected with the same serotype (*S*. Typhimurium).

### Transition parameters and *R*_*0*_ values

Note that the stochastic SIR model presented here is only a discrete-time approximation to the real transmission dynamics, i.e. limited to bi-weekly intervals. In particular, when the number of susceptible animals is small and the infection intensity high, then the expected number of infectious animals will tend to be overestimated [5].

The estimate of the transition rate β (from S to I) is low compared with other infectious diseases (such as swine influenza) and reflects the fact that in most of the herds, *Salmonella* does not cause outbreaks but maintains a residual level of infection represented by infectious and carrier animals that enable the infection to persist in the herds. The credible interval for the transition parameter α (from I to R), matches the variability of shedding duration that is known from experimental and field studies [13,39]. The transition rate estimate ν (from R to I) is small and possibly dependent on stress events (the authors of the Kranker *et al*. study [10] report that two cohorts in which animals began shedding in a second round had a slurry overflow which can be considered a stressful event). The variance of the cohort time-dependent random effect was high and a possible explanation for that is the different management of cohorts which in turn induces high variability (between cohorts) in the transmission data. In future studies this should be taken into consideration as a way to minimize transmission of infection.

For spread to occur, *R*_*0*_ should be above one. Looking at Figure 5 we can see that there is high probability that *R*_*0*_ > 1, 94% specifically. The median *R*_*0*_ value was 1.91 indicating that *Salmonella* Typhimurium was spreading in most of the cohorts. The value is not high (third quartile of *R*_*0*_ is less than three) implying it would not spread rapidly through the susceptible populations under management systems similar to the ones used in these herds. With lower probability, *R*_*0*_ is high enough to cause outbreaks, e.g. probability that *R*_*0*_ > 5 is 2.5%.

The *R*_*0*_ 95% Credible Interval (CrI) ranges from 0.78 to 5.24. The higher values reflect that animals infected with a high infectious dose have a longer shedding period [13] than the ones infected with low infectious dose, and so the former can cause an outbreak. This makes sense because *Salmonella* Typhimurium is an agent that primarily spreads via the faecal-oral route.

Few studies are available to aid in defining infectious animals, but the experimental and field studies conducted by some authors [13,15] support the duration of infectiousness used in our study.

A next step in our investigation will be to include the estimated transmission parameters (β, α, ν) in a stochastic simulation model developed by the authors to simulate the spreading of *Salmonella* Typhimurium in swine herds and thus test the effectiveness of different control strategies.

## Conclusions

A Bayesian framework was proposed, to estimate *Samonella* Typhimurium transmission parameters, and has been successfully implemented using data from Danish pig herds. The model extends the current established methodology of utilising GLMs to implement stochastic SIR models. Random effects were added to i) explicitly allow for cohort heterogeneity in the data (i.e. allow for the fact that pigs were grouped in cohorts), ii) capture possible unobserved cohort effects and iii) avoid the problem of overdispersion. Results in terms of posterior samples allow for direct probabilistic statements about model parameters, which may be also used in other analyses such as simulation models for testing management strategies.

The issue of underestimating infectious pigs due to testing sensitivity was addressed by predicting the number of non-detected pigs, using i) prior information about test sensitivity and ii) the observed data. In doing that, the probability of non-detection was treated as an unknown parameter which was estimated at the same time as the transmission parameters.

All model unknowns (transmission parameters, cohort random effects, non-detected pigs, probability of non-detection) were estimated simultaneously, implying that all possible sources of uncertainty were modelled, in turn giving more confidence about the estimates of the transmission parameters.

## Competing interests

The authors declare that they have no competing interests.

## Authors’ contributions

CCG and TE were involved in the design and performed the statistical modelling analysis and drafted the manuscript. TB was involved in the design of the statistical analysis and the revision of the manuscript for intellectual content. LA and PB were involved in the revision of the manuscript for intellectual content. JNR was involved in the drafting and revision of the manuscript for intellectual content. All authors approved the final manuscript.
